# The Effects of Different C/N Ratios on Rhizosphere Soil Environment of *Lonicera caerulea* L.

**DOI:** 10.3390/plants15203199

**Published:** 2026-10-19

**Authors:** Jinying Li, Yali Zhang, Zhengle Yin, Rui Li, Zihao Li, Shanshan Xu, Yulin Pan, Yanan Li, Lin Wu, Ying Wang

**Affiliations:** College of Horticulture, Jilin Agricultural University, Changchun 130118, China; 18363242830@163.com (Y.Z.); yzl20020201@163.com (Z.Y.); 17661788597@163.com (R.L.); 13067786156@163.com (Z.L.); youzi_512mob_003@163.com (S.X.); 15804829829@163.com (Y.P.); liyanan@jlau.edu.cn (Y.L.); linw@jlau.edu.cn (L.W.)

**Keywords:** *Lonicera caerulea* L.var. *edulis* Turcz. ex Herder, C/N, soil nutrients, soil microorganisms, soil enzyme activity

## Abstract

Aiming to solve the problems of excessive chemical fertilizer application and reduced soil fertility in *Lonicera caerulea* cultivation, this study investigated soil carbon and nitrogen nutrients, enzyme activities, and rhizosphere microbial characteristics using potted seedlings. Seven treatments, including control, single straw application, single nitrogen fertilization, and four C/N ratios (10, 15, 20, 25), were established. The results showed that a soil C/N ratio of 15–25 achieved the optimal soil fertilization effect. The 20:1 C/N ratio produced the highest soil total nitrogen and active organic carbon contents, as well as the maximum nutrient-converting enzyme activity. C/N ratio significantly regulated rhizosphere microbial communities: increasing nitrogen improved bacterial diversity but slightly reduced fungal diversity, and C/N imbalance altered dominant fungal phyla. Soil nitrogen and active organic carbon were positively correlated with bacterial diversity and negatively correlated with fungal diversity. Based on a comprehensive analysis of various indicators, a C/N ratio range of 15:1 to 25:1 improves soil fertility, while a ratio of 20:1 yields the most balanced overall results, enhancing soil carbon and nitrogen reserves, optimizing the microbial community, and reducing nutrient loss. This study provides a reference for fertilizer reduction and efficiency improvement in *Lonicera caerulea* cultivation.

## 1. Introduction

*Lonicera caerulea* is a small berry-bearing tree species of significant economic value; its fruits are rich in functional compounds such as anthocyanins and polyphenols and possess high nutritional and health benefits [1]. In recent years, with the restructuring of the agricultural industry and the rise in specialty fruit and forestry industries, the cultivation of *Lonicera caerulea* has continued to expand in cool and temperate regions such as Northeast, Northwest, and North China, becoming an important crop that drives rural revitalization and the development of regional specialty economies [2]. However, the cultivation and management of *Lonicera caerulea* are still largely guided by traditional experience. In terms of soil fertility management, issues such as excessive application of chemical fertilizers and insufficient use of organic matter have led to nutrient imbalances and a decline in soil fertility, which not only affects fruit quality but also hinders steady increases in yield [3]. Therefore, exploring scientific and efficient soil nutrient management measures is of great significance for achieving the green and sustainable development of the *Lonicera caerulea* industry.

As an important measure for improving soil fertility, straw return to the field plays a crucial role in improving soil physicochemical properties, increasing organic matter content, and promoting nutrient cycling [4]. Straw is rich in carbon but has a low nitrogen content; when returned to the field, microorganisms tend to compete with crops for available nitrogen, which in turn affects crop growth [5]. By adjusting the carbon-to-nitrogen ratio during straw incorporation, it is possible to effectively regulate the processes by which microorganisms fix and mineralize soil nitrogen, thereby optimizing the soil’s nutrient supply capacity [6]. An appropriate C/N ratio not only accelerates straw decomposition and promotes the accumulation of soil organic carbon, but also synergistically regulates soil enzyme activity and enhances soil biological fertility [7]. Currently, research on regulating the C/N ratio through straw return to the field has been widely conducted for both food crops and cash crops, confirming that the rational application of nitrogen sources to adjust the C/N ratio is an effective way to improve fertilizer use efficiency and reduce chemical fertilizer application rates.

Soil fertility is the foundation for high crop yields and quality, and the C/N ratio, as a key indicator of soil organic matter quality and nutrient cycling, directly influences soil microbial activity, nutrient availability, and enzyme system function [8]. Studies have shown that the optimal C/N ratio created by the combined application of exogenous organic matter and nitrogen can modulate the structure and function of microbial communities, alter the activity of key enzymes involved in soil carbon and nitrogen metabolism (such as urease, sucrase, and catalase), and thereby drive the transformation and release of soil nutrients [9]. An appropriate C/N ratio range helps establish a “carbon-nitrogen synergistic” soil microecosystem, enabling a dynamic balance between nitrogen supply and crop demand. This approach ensures a sufficient supply of nutrients to crops while reducing nitrogen loss, in line with the green development philosophy of “reducing fertilizer use while increasing efficiency” [10]. However, there is currently little research on the role of *Lonicera caerulea* in regulating soil fertility through the C/N ratio, and there is a lack of systematic, quantitative theoretical foundations and technical parameters.

In light of this, this study used potted *Lonicera caerulea* seedlings as the research subjects. By applying treatments with different C/N ratio gradients, the study systematically investigated their effects on soil nutrient content and soil microbial diversity to determine the optimal C/N ratio range for *Lonicera caerulea* cultivation. The research findings aim to elucidate the underlying mechanisms by which the C/N ratio regulates soil fertility in *Lonicera caerulea* cultivation. They provide a scientific basis for establishing a soil-targeted fertilization technology system based on the resource utilization of crop straw. This research holds significant theoretical and practical implications for promoting standardized *Lonicera caerulea* cultivation, facilitating the efficient utilization of agricultural waste, and achieving reduced chemical fertilizer application while enhancing fertilizer efficiency.

## 2. Results

### 2.1. Effects of Different C/N Ratios on Soil Nitrogen and Carbon Fractions in Lonicera caerulea L.var. edulis Turcz. ex Herder Cultivation

Different fertilization treatments had a significant effect on soil organic matter, total nitrogen, activated carbon, and nitrogen content at various levels (Table 1). All carbon and nitrogen parameters in the CK group were the lowest among all groups. The levels of soil organic matter (SOM), total nitrogen (TN), alkali-hydrolyzable nitrogen (AN), readily oxidizable carbon (ROC), dissolved organic carbon (DOC), ammonium nitrogen (NH_4_^+^-N), and nitrate nitrogen (NO_3_^−^-N) were significantly lower than those in most treatments combining straw and nitrogen fertilizer. The SN series treatments, which combined straw with nitrogen fertilizer, showed overall better carbon and nitrogen nutrient status than the CK, S, and N single treatments, with significant gradient differences in various indicators depending on the mixing ratio. TN, AN, NH_4_^+^-N, and NO_3_^−^-N all reached their maximum levels in the SN3 treatment, significantly higher than in the other treatment groups. As the mixing ratio increased from SN1 to SN3, the soil’s total nitrogen, activated carbon, and available nitrogen contents rose steadily, but nutrient levels began to decline in the SN4 treatment. In terms of SOM, the N and SN3 treatments had significantly the highest levels, while the CK, S, SN1, and SN2 treatments had relatively low levels of SOM, and the SN4 treatment fell in the middle range. Among the active organic carbon components, ROC and DOC were generally higher under the straw-nitrogen combination treatments, with DOC in the SN3 and SN4 treatments significantly higher than in the CK and N treatments. NO_3_^−^-N was the most sensitive to the application ratio, and the NO_3_^−^-N content in the SN series treatments was significantly higher than that in the treatments involving straw alone or nitrogen fertilizer alone. The trends in AN and NH_4_^+^-N were generally consistent with those of NO_3_^−^-N.

### 2.2. Effects of Different C/N Ratios on Soil Enzyme Activity in Lonicera caerulea L.var. edulis Turcz. ex Herder

There were significant differences in the activities of soil urease(URE), sucrase(SUC), catalase(CAT), acid phosphatase(ACP), and β-glucosidase(β-GC) among the different treatment groups (Table 2). Overall, the activity of various soil enzymes in the CK group was the lowest; specifically, the activities of URE, SUC, CAT, and ACP were significantly lower than those in all treatments where nitrogen fertilizer was applied in combination with straw, and the activity of β-GC was significantly lower than that in all treatment groups. S and N treatments only slightly increased the activity of various enzymes. Most enzyme parameters showed no significant difference from the CK, with the exception of β-GC activity, which was significantly higher than that of the CK. In all treatments combining straw with nitrogen fertilizer, soil enzyme activity was significantly higher overall than in the CK, S, and N treatments alone. The activity of all five soil enzymes peaked in the SN3 treatment and was significantly higher than in all other groups. From SN1 to SN3, the activity of various soil enzymes showed a steady upward trend; by the SN4 treatment, enzyme activity had decreased to varying degrees. In terms of URE activity, SN3 was significantly the highest; S, SN1, SN2, and SN4 were at the same level, and N and CK had the lowest activity. The patterns of change in SUC and β-GC were consistent; SN2, SN3, and SN4 activities were significantly higher than those of CK, S, and N, with the maximum values for all occurring at SN3. The CAT activity increased gradually as the application rate increased, with SN3 showing significantly higher activity than the other treatments; for ACP, only SN3 reached a significantly higher level, while differences among the other treatments were minor.

### 2.3. Analysis of Rhizosphere Microbial Diversity in Lonicera caerulea L.var. edulis Turcz. ex Herder

**Figure 1:**
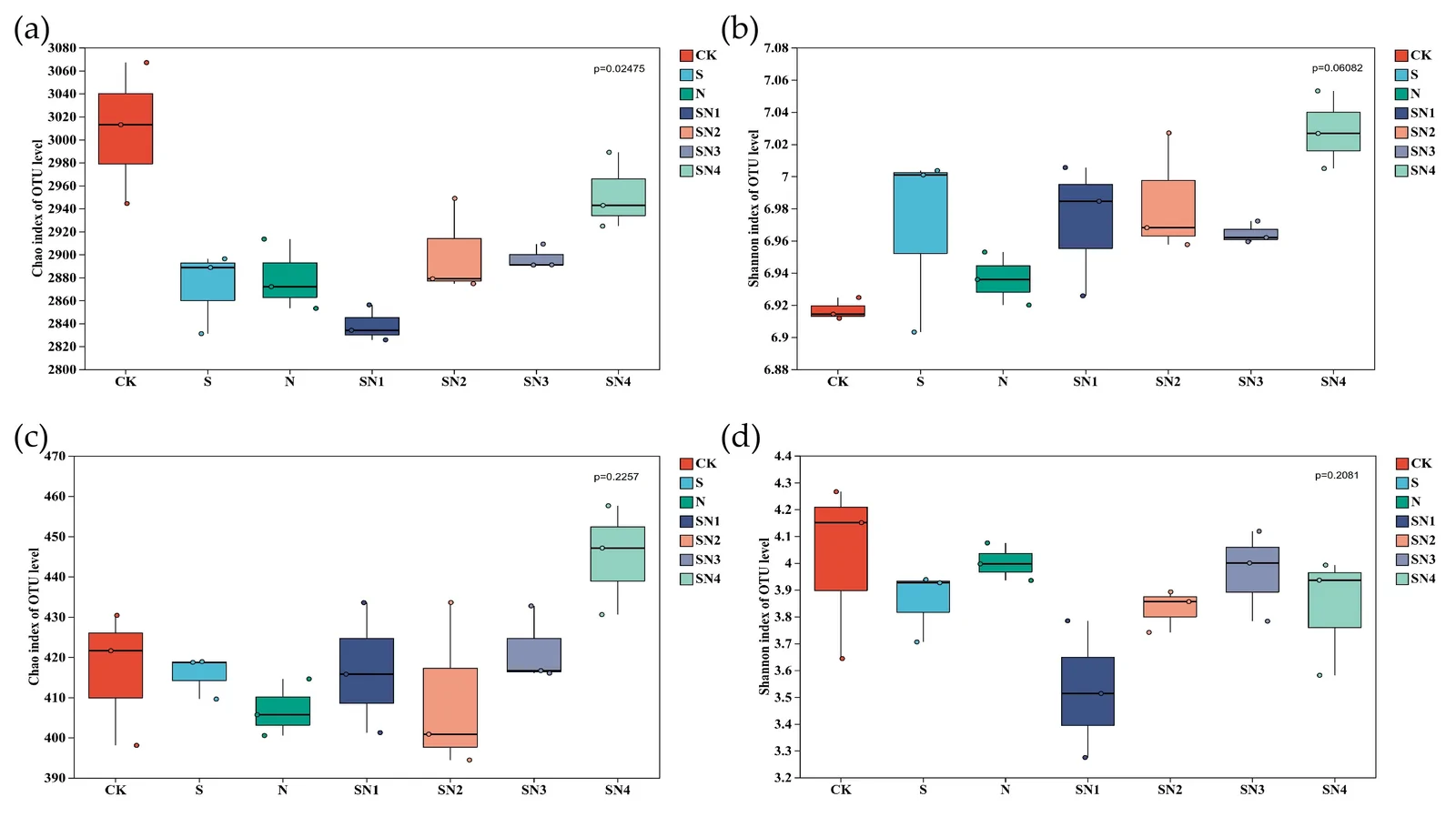
Box plots of bacterial and fungal α-diversity in the rhizosphere soil of *Lonicera caerulea*. Note: (**a**,**b**) Bacteria; (**c**,**d**) Fungi.

Under the influence of different C/N ratios of straw and nitrogen fertilizer application, significant differences were observed in the Chao-1 index at the bacterial OTU level among treatment groups (Figure 1a). Bacterial richness in the CK treatment was significantly higher than in the other treatments, with reductions of approximately 4.7%, 5.0%, 6.0%, 4.0%, 3.7%, and 2.7%, respectively, compared to CK. Differences in the Shannon index at the bacterial OTU level among treatment groups were near the level of statistical significance (Figure 1b). The Shannon index for all straw treatments and straw-nitrogen fertilizer combination treatments was higher than that of the CK treatment, with the SN4 treatment reaching a peak, showing an increase of approximately 1.7% compared to CK. Other treatments also exceeded CK, but the differences between groups did not reach statistical significance. The study indicates that straw return combined with nitrogen fertilization can alter the structure of the soil bacterial community by regulating the rhizosphere carbon-nitrogen balance. Applying straw or urea alone results in a significant decrease in bacterial species richness, while the SN4 treatment with a high C/N ratio demonstrates a more pronounced effect on enhancing bacterial species diversity. The combined application of carbon and nitrogen elicited a gradient response in fungal community α-diversity, but differences between groups were not statistically significant (Figure 1c,d); the Chao-1 index at the fungal OTU level was lowest in the N and SN2 treatments, while the SN4 treatment was higher than CK and reached the peak among all groups; the Shannon index at the fungal OTU level was relatively high in the CK treatment, whereas the SN1 treatment exhibited an extremely low value.

### 2.4. Analysis of the Rhizosphere Microbial Community Structure of Lonicera caerulea L.var. edulis Turcz. ex Herder

**Figure 2:**
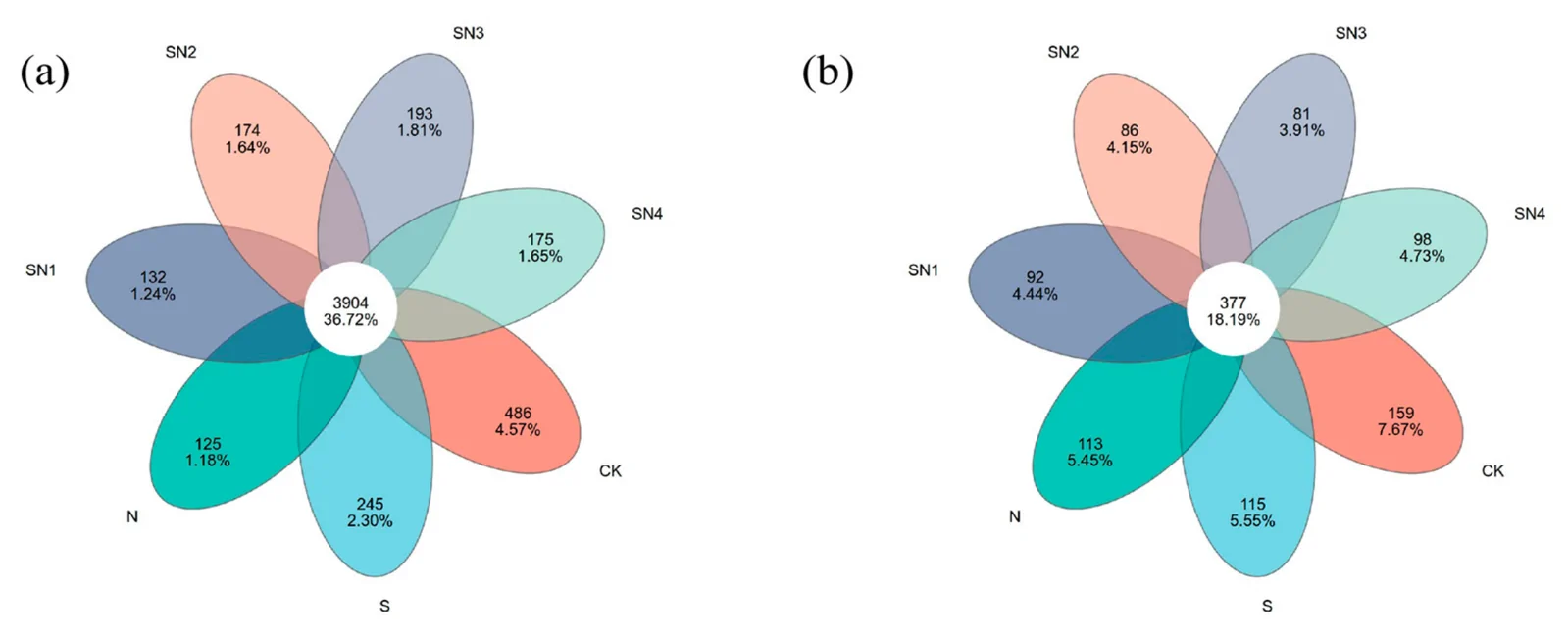
Venn diagram showing the OTU levels of bacteria and fungi in the rhizosphere soil of *Lonicera caerulea*. Note: (**a**) Bacteria; (**b**) Fungi.

Under different C/N ratio treatments, there were significant differences in the Venn diagram results for OTUs of rhizosphere bacteria (Figure 2a) and fungi (Figure 2b) in *Lonicera caerulea*. The analysis results indicate that the *Lonicera caerulea* rhizosphere bacterial community (Figure 2a) under different C/N ratio treatments contained 3904 shared OTUs, accounting for 36.72% of the total OTUs; this high proportion of shared bacterial communities suggests greater stability in the bacterial community structure. The CK group had 486 unique bacterial OTUs (4.57%), the highest among all groups. The groups treated with straw (S) or urea (N) alone had 245 (2.30%) and 125 (1.18%) unique OTUs, respectively; the application of nitrogen fertilizer alone had the strongest inhibitory effect on unique bacterial species. In the mixed-application groups SN1, SN2, and SN3, the number of unique bacterial OTUs gradually increased with rising C/N ratios, reaching 193 (1.81%) in SN3, before declining to 175 (1.65%) in SN4. It can therefore be concluded that increasing the C/N ratio can, to some extent, mitigate the depletion of unique bacterial taxa caused by nitrogen fertilizer, and that the modification effect of straw application alone on the bacterial community is stronger than that of urea application alone. The fungal community (Figure 2b) had a total of 377 core OTUs, accounting for 18.19% of the total fungal OTUs. The 159 unique fungal OTUs (7.67%) in the CK treatment also represented the highest value among all groups, while the S and N treatments had 115 (5.55%) and 113 (5.45%) unique OTUs, respectively; the SN3 treatment had the fewest unique fungal OTUs, at 81 (3.91%).

**Figure 3:**
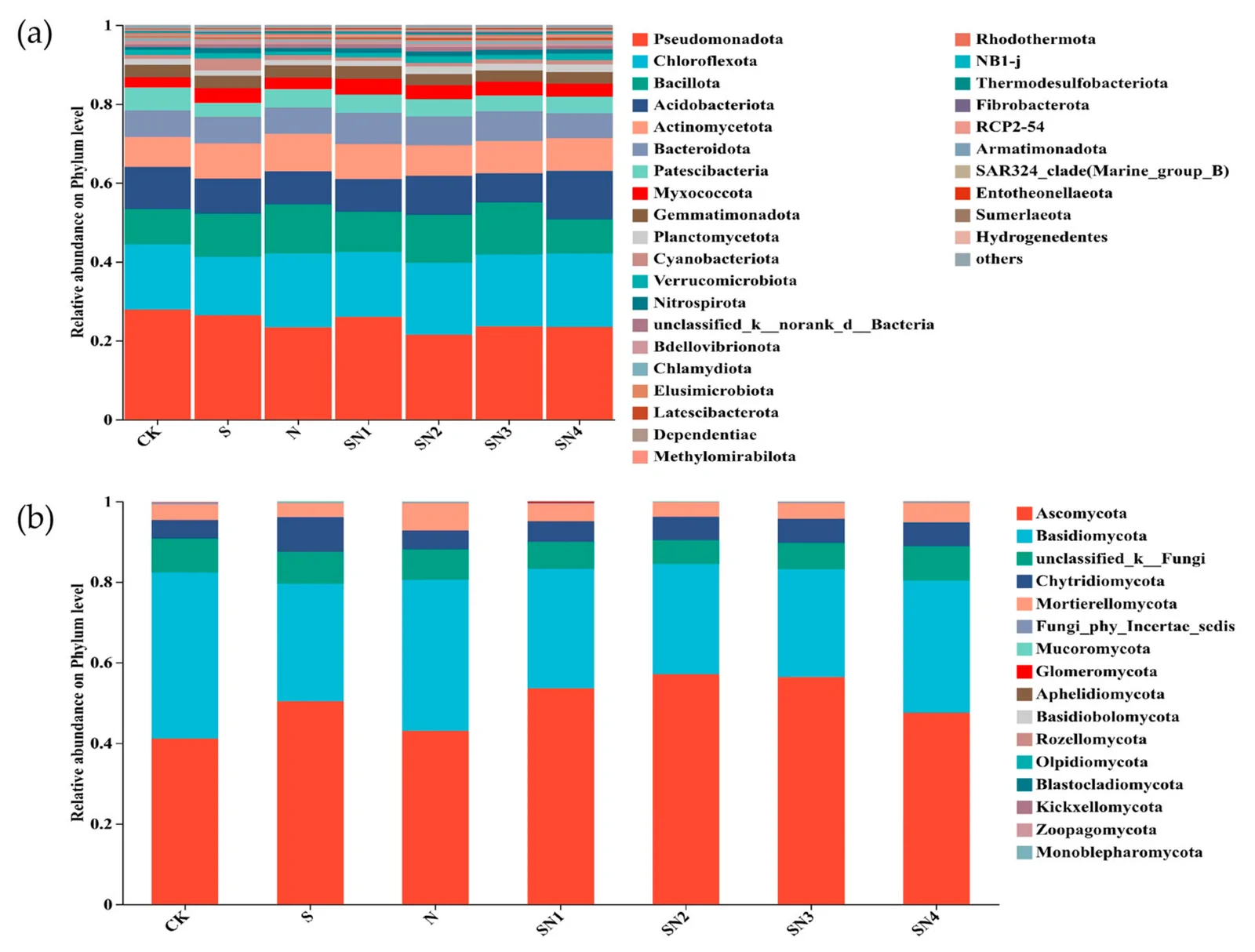
Distribution of relative abundances of the rhizosphere microbial community of *Lonicera caerulea* at the phylum level. Note: (**a**) Bacteria; (**b**) Fungi. The relative abundance for each sample was calculated as a percentage of the total valid sequences, which were classified using the RDP classifier. “Others” represents the sum of relative abundances at the phylum level for all taxa not included in the top 30.

The results of the stacked bar charts showing the relative abundance of phylum-level species indicate that the dominant phyla in the soil bacterial communities (Figure 3a) across all treatments remained relatively stable. *Pseudomonadota* accounted for the highest proportion in all treatments, followed by *Chloroflexota*, *Bacillota*, and *Acidobacteriota*. The relative abundances of these dominant bacterial phyla did not fluctuate dramatically among the CK, S, N, and SN1–SN4 treatments, exhibiting only slight gradient differences in response to changes in the C/N ratio. The proportions of other rare bacterial phyla were extremely low and distributed uniformly across all groups. In contrast, the species composition at the phylum level of the fungal community (Figure 3b) was significantly influenced by different C/N ratios. *Ascomycota* and *Basidiomycota* are the dominant fungal phyla. In the CK treatment, the abundance of *Ascomycota* was lower than that of *Basidiomycota*. In the treatments involving sole application of straw (S), sole application of nitrogen fertilizer (N), and combined application of SN1–SN3, the relative abundance of *Ascomycota* increased significantly and exceeded that of *Basidiomycota*. Specifically, the abundance of *Ascomycota* reached its peak in the SN2 and SN3 treatments. In the SN4 treatment, the abundance of *Ascomycota* declined somewhat and the proportion of *Basidiomycota* rebounded. Minor fungal phyla such as *Mortierellomycota* and *Chytridiomycota* remained stable across all treatment groups but at relatively low levels.

### 2.5. Analysis of Rhizosphere Soil Microbial Community Structure Based on PCoA of Lonicera caerulea L.var. edulis Turcz. ex Herder

**Figure 4:**
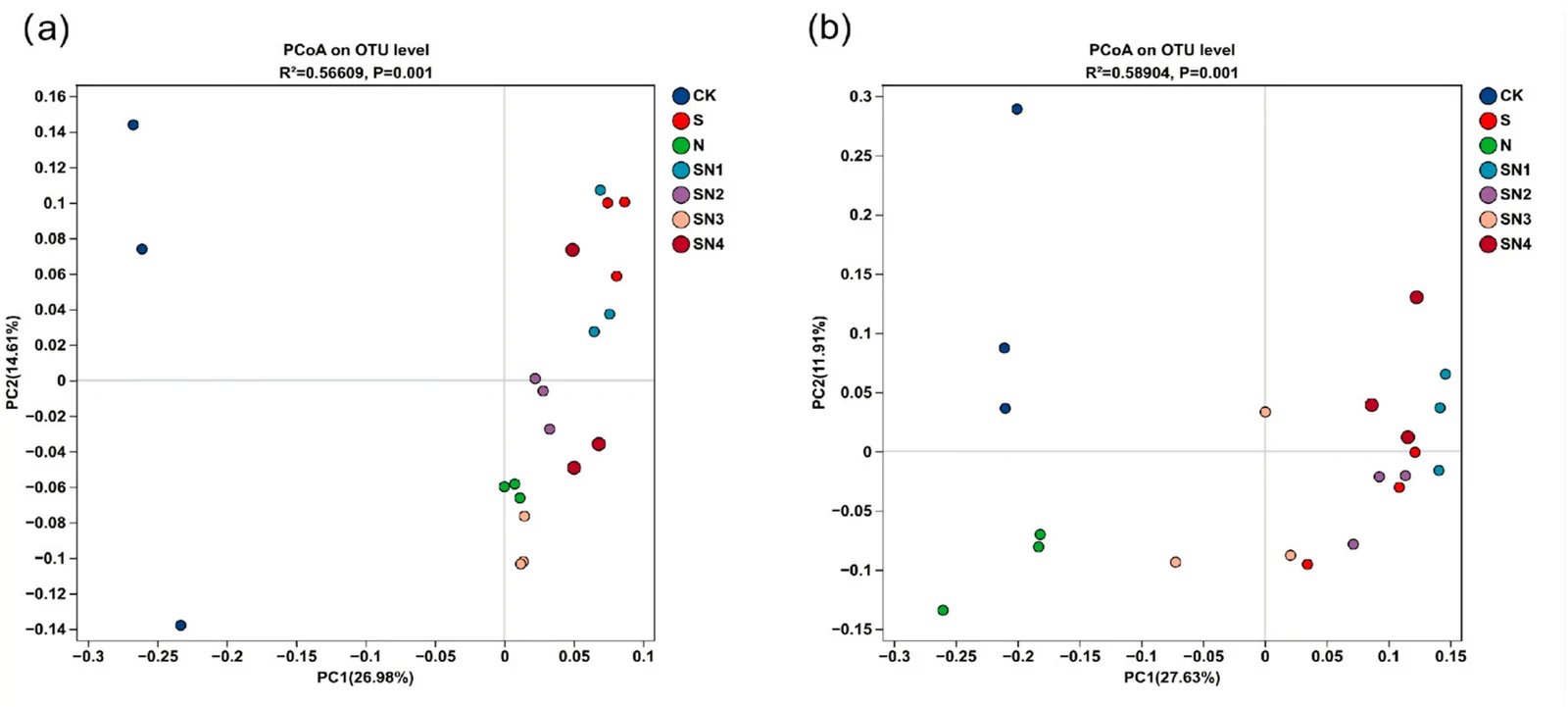
PCoA of bacterial (**a**) and fungal (**b**) communities at the OTU level based on Bray–Curtis distance. Values in each panel indicate PERMANOVA R^2^ and *p*-value.

Principal coordinate analysis (PCoA) based on Bray–Curtis distances at the OTU level, combined with a PERMANOVA test, indicated that bacterial communities (pseudo-F = 3.044, R^2^ = 0.566, *p* = 0.001; Figure 4a) and fungal communities (pseudo-F = 3.344, R^2^ = 0.589, *p* = 0.001; Figure 4b) showed significant differences. Specifically, PC1 and PC2 explained 26.98% and 14.61% of the total variance in the bacterial community, respectively, while PC1 and PC2 explained 27.63% and 11.91% of the total variance in the fungal community, respectively. Along the PC1 axis, all straw return treatments (S, SN1–SN4) were clearly separated from the no-straw controls (CK, N), indicating that maize straw return may be the primary factor driving the differentiation of rhizosphere bacterial and fungal communities. Within the straw return treatments, as the urea application rate increased, the communities gradually deviated from CK along the secondary axis, suggesting that the carbon-to-nitrogen ratio may further regulate the direction of microbial succession during the straw decomposition process. Notably, the SN3 treatment (C/N = 20) occupied an intermediate position in the ordination plot: its bacterial community was closer to that of the treatment with nitrogen fertilizer alone, a phenomenon that may reflect the relatively complete release of mineral nitrogen at this ratio; while its fungal community was closest to CK among all straw return treatments, possibly indicating relatively moderate fungal decomposition activity without any obvious fungal community outbreaks. It should be noted that the above interpretations regarding community function and ecological processes are inferred from the clustering results and require further validation using functional gene annotation or extracellular enzyme activity data. The intermediate distribution pattern exhibited by SN3 in the sequencing space may be related to the relative coordination of straw decomposition rate and nitrogen supply levels under this C:N ratio; however, it should not be defined as the “optimal” ratio based solely on differences in community structure.

### 2.6. Analysis of the Correlation Between Rhizosphere Soil Microbial Diversity and Soil Environmental Factors of Lonicera caerulea L.var. edulis Turcz. ex Herder

**Figure 5:**
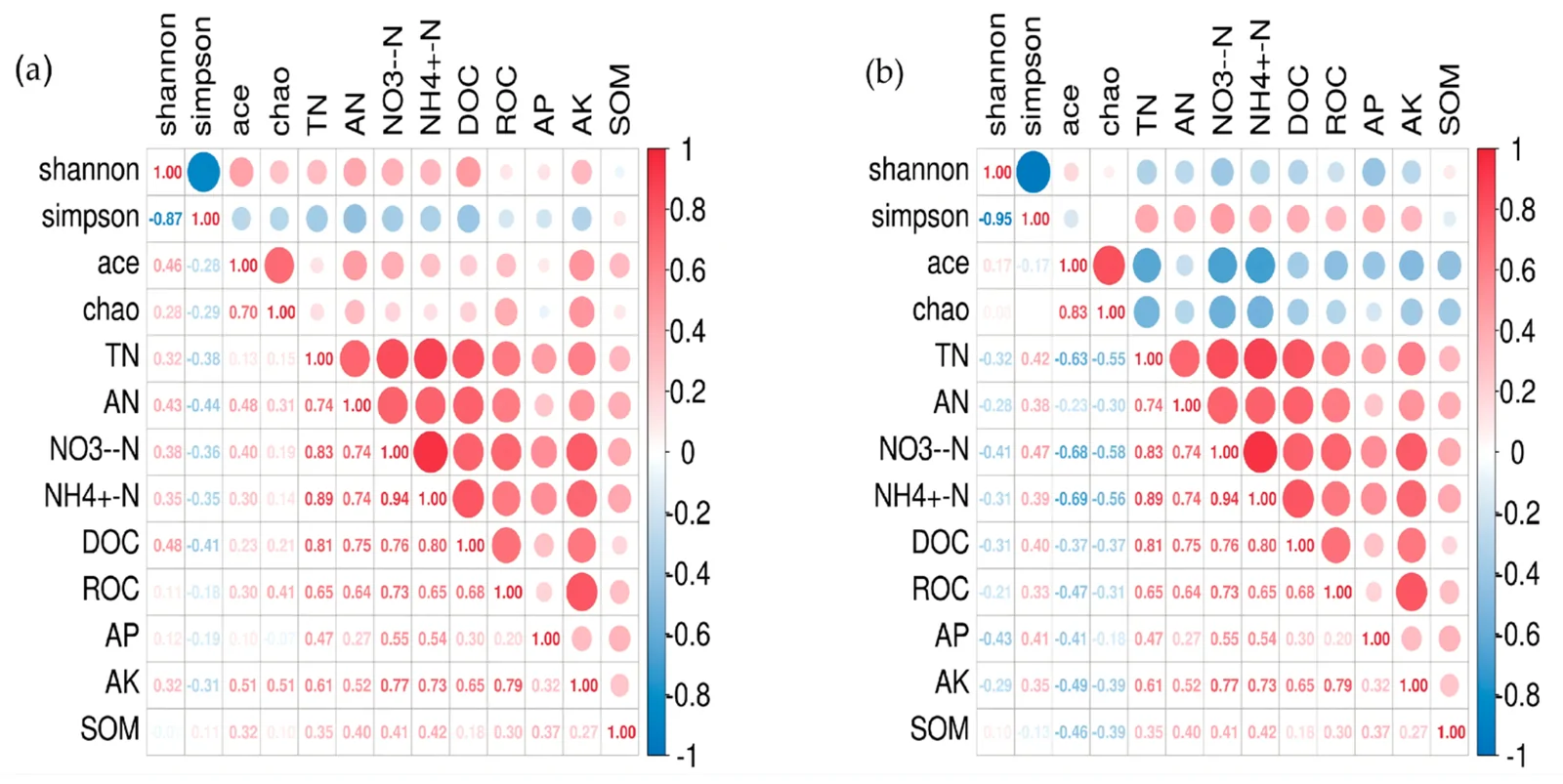
Analysis of the correlation between microbial diversity in the rhizosphere soil of *Lonicera caerulea* and soil environmental factors. Note: (**a**) Bacteria; (**b**) Fungi. TN: Total nitrogen; AN: Alkaline-hydrolyzable nitrogen; NO_3_^−^-N: Nitrate nitrogen; NH_4_^+^-N: Ammonium nitrogen; DOC: Dissolved organic carbon; ROC: Readily oxidizable carbon; AP: Olsen-P; AK: available potassium; SOM: Soil organic matter.

Pearson correlation analysis revealed that, under different C/N ratio treatments, the Shannon index of soil bacteria (Figure 5a) was significantly negatively correlated with the Simpson index, while the Ace index was significantly positively correlated with the Chao1 index. The bacterial ACE, Chao-1, and Shannon indices all showed positive correlations with total nitrogen, alkali-hydrolyzable nitrogen, nitrate nitrogen, ammonium nitrogen, and dissolved organic carbon. This indicates that increased soil nitrogen content can sustain higher bacterial community diversity and species richness, whereas the Simpson index was negatively correlated with various nitrogen indicators, suggesting that increased nutrient levels reduced the dominance of the bacterial community. Analysis revealed that for soil fungi (Figure 5b), the Shannon index showed a stronger negative correlation with the Simpson index, while the ACE and Chao-1 indices were also significantly positively correlated. The Shannon index for fungi was negatively correlated with total nitrogen, alkali-hydrolyzable nitrogen, nitrate nitrogen, and ammonium nitrogen, with the ACE and Chao-1 richness indices showing a stronger negative correlation with nitrogen. This indicates that nitrogen enrichment significantly inhibits the species richness and diversity of fungal communities. At the same time, the fungal Shannon index showed a moderate negative correlation with Olsen-P, while bacterial diversity exhibited only a weak correlation with total phosphorus, indicating that fungal communities are more sensitive to changes in soil phosphorus. Neither bacterial nor fungal diversity indices showed a significant correlation with soil organic matter. Furthermore, strong positive correlations were observed among various nitrogen components in the soil; dissolved organic carbon and readily oxidizable carbon exhibited trends synchronous with nitrogen changes, while Olsen-P, available potassium, organic matter, and the carbon-to-nitrogen ratio showed relatively low correlations.

## 3. Discussion

Beyond its regional importance in northeast China, *Lonicera caerulea* (haskap/honeyberry) is an emerging boreal berry crop of growing international significance. Domesticated in Russia since the mid-twentieth century and subsequently advanced through breeding programs in Canada, Japan, and northern Europe [11], haskap has recently expanded into commercial plantations across North America and northern Eurasia. Nevertheless, belowground nutrient management in haskap plantations remains poorly characterized, in stark contrast to better-studied temperate berries such as blueberry (*Vaccinium* spp.), where nitrogen form, soil pH, and ericoid mycorrhizal associations have long been recognized as central to nutrient acquisition [12,13]. Against this background, the present study combined straw return with nitrogen fertilization and precisely regulated the soil C/N ratio, thoroughly examining the effects of different C/N ratios on nitrogen and carbon fractions, enzyme activity, and microbial community structure and diversity in potted soil for *Lonicera caerulea*.

By combining straw return to the field with nitrogen fertilizer application and precisely regulating the soil C/N ratio, this study thoroughly examined the effects of different C/N ratios on nitrogen and carbon fractions, enzyme activity, and microbial community structure and diversity in potted soil for *Lonicera caerulea*. The study found that when the soil C/N ratio was adjusted to 20:1, the comprehensive soil fertility effect peaked. This finding not only aligns with the pattern observed by Li Xueli et al. [14] in tobacco cultivation—where a C/N ratio of 24–28 was most conducive to the coordination of plant carbon and nitrogen metabolism—but also closely corresponds with the conclusion by Ge Shunfeng et al. [15] in apple orchards, which determined that nitrogen use efficiency is optimal within a C/N ratio range of 15–25. This convergence across tobacco, apple, and *Lonicera caerulea* supports a broadly conserved stoichiometric optimum for woody perennial nutrient cycling, consistent with international orchard-system studies showing that balancing carbon inputs with nitrogen supply is a universal lever for improving nitrogen use efficiency in temperate fruit plantations [16,17]. It thus reveals the sensitivity and universal patterns of soil C/N ratios in the rhizosphere of woody plants.

From the perspective of micro-regulatory mechanisms, corn stover itself has a high C/N ratio. Under single-nitrogen-supply conditions, nitrogen limitation is prone to trigger microbial growth, forcing microorganisms to extensively assimilate soil mineral nitrogen to meet the demand for biomass synthesis, resulting in a significant short-term biogeochemical retention effect and temporarily reducing the supply of soil available nitrogen [6]. Applying nitrogen fertilizer alone can temporarily increase the inorganic nitrogen content, but without sufficient easily decomposable carbon sources as energy substrates, the proliferation of microorganisms is restricted, and they are unable to effectively retain the excess nitrogen through biological fixation. Mineral nitrogen is prone to loss through leaching, volatilization, etc., which is not conducive to the stable accumulation of the total nitrogen pool in the soil [18]. The scientific combination of crop straw and nitrogen fertilizer can adjust the substrate C/N ratio to a range suitable for microorganisms, achieving a dynamic balance between nitrogen mineralization and biological retention: an adequate carbon source provides energy to drive microbial metabolism, promoting the assimilation and retention of nitrogen and significantly reducing nitrogen loss; an appropriate nitrogen supply relieves nitrogen limitation on microorganisms, accelerates the decomposition of crop straw organic matter and the mineralization of organic nitrogen, and ultimately achieves a simultaneous increase in both the soil’s total nitrogen pool and its supply of available nitrogen [19,20]. In this study, the decrease in total nitrogen and inorganic nitrogen content under high C/N ratio treatments was precisely due to nitrogen deficiency, which reduced microbial decomposition efficiency and slowed straw decomposition; under low C/N ratios, nitrogen excess exacerbated the risks of nitrification and leaching, which is detrimental to long-term nutrient accumulation, further confirming the stoichiometric principles of carbon-nitrogen coupling and transformation. Soil active organic carbon fractions are also significantly regulated by the C/N ratio; soluble carbon and easily oxidizable carbon primarily originate from decomposition intermediates and microbial metabolic secretions, directly reflecting the intensity of organic matter transformation [21]. In this study, both types of active carbon and total organic carbon were highest under the C/N = 20:1 treatment, indicating that microbial metabolic activity is strongest at an optimal carbon-to-nitrogen ratio, straw decomposition produces the most active carbon components, and the accumulation of metabolic products also promotes humus formation. An excessively low C/N ratio is detrimental to organic carbon sequestration; conversely, an excessively high C/N ratio weakens microbial catabolism and reduces the release of active carbon. In summary, regulating the C/N ratio to achieve a balance between carbon and nitrogen substrates is the key strategy for simultaneously enhancing the effectiveness of soil carbon and nitrogen pools through straw return to the field. This mechanism is not specific to *Lonicera caerulea*: international work on deep-rooted perennial systems has likewise shown that coupled carbon and nitrogen supply is essential for microbial stabilization of soil organic matter, particularly in the deep and stratified rooting zones that characterize perennial—rather than annual—cultivation [22]. This approach also optimizes microbial community structure and enzyme activity, thereby enhancing the multifunctionality of the soil ecosystem.

As key catalysts for biochemical reactions, soil enzymes’ activity directly reflects the intensity of soil nutrient cycling and the functional status of microorganisms. This study found that the application of straw or nitrogen fertilizer alone had only a limited effect on increasing the activity of urease, β-glucosidase, and acid phosphatase, whereas the combined application of straw and nitrogen fertilizer significantly enhanced enzyme activity. This enhancement followed a trend of initially increasing and then decreasing as the C/N ratio rose, peaking at a C/N ratio of 20:1. This finding is highly consistent with the pattern observed by Li Xueli et al. [14] in flue-cured tobacco cultivation, where an optimal C/N ratio significantly increased the activity of key enzymes involved in carbon and nitrogen metabolism, while an imbalance led to a decrease in activity. It is also in line with international orchard-floor-management studies, in which straw and living-mulch treatments markedly raised soil sucrase, β-glucosidase, and other carbon-cycling enzymes relative to clean cultivation in peach and pear orchards [23,24]. The study found that the response patterns of different enzyme classes varied to some extent: sucrase activity stabilized when C/N ≥ 15:1, indicating its high sensitivity to carbon source input; catalase activity continued to increase with rising C/N ratios, possibly related to the increased proportion of fungal communities and the heightened demand for the degradation of recalcitrant substrates under high C/N conditions [25]. Overall, the evolution of soil enzyme activity closely aligns with the patterns of carbon and nitrogen accumulation, further confirming that C/N = 20:1 is the optimal stoichiometric equilibrium point for coordinating enzymatic processes in the rhizosphere’s carbon, nitrogen, and phosphorus cycles, thereby providing a theoretical basis for optimizing the biochemical functions of agricultural soils.

Soil microorganisms are the key agents in straw decomposition and nutrient transformation, and their community structure and diversity are highly sensitive to changes in the substrate C/N ratio. Previous studies have shown that the carbon-to-nitrogen composition of soil substrates is a key factor shaping microbial communities, and changes in the C/N ratio can selectively favor microbial groups with different nutritional strategies [26]. This study found that exogenous C/N inputs significantly regulate bacterial α-diversity but have no significant effect on fungal α-diversity; bacterial and fungal species response patterns to fluctuations in the C/N ratio show marked differences. Bacterial community characteristics differed significantly: the blank control group had the highest bacterial richness and relatively low evenness, with the community dominated by oligotrophic groups; following the addition of straw and nitrogen fertilizer, eutrophic bacterial populations proliferated extensively, which may inhibit the growth of oligotrophic groups and may contribute to a slight decrease in bacterial richness, an increase in evenness, and an overall rise in the Shannon index. As the C/N ratio increased, the bacterial Shannon index continued to rise, peaking in the SN4 treatment with a high C/N ratio. A high-carbon and low-nitrogen environment may support the coexistence of various functional bacterial communities by providing diverse carbon sources. Meanwhile, the low-nitrogen condition may avoid the loss of sensitive species associated with high-nitrogen stress and may help maintain the diversity of bacterial communities [27,28,29]. Fungal communities exhibited greater tolerance to fluctuations in the C/N ratio, with no significant differences in α-diversity among groups; however, their community structure at the phylum level responded more sensitively. The abundance of the Ascomycota phylum first increased and then decreased as the C/N ratio rose, peaking under medium-to-high C/N ratio treatments. These saprotrophic fungi may efficiently decompose readily degradable organic carbon and may dominate when substrates are abundant. However, nitrogen limitation associated with a continuously rising C/N ratio inhibits their growth, while the competitiveness of Basidiomycota—which specialize in lignin decomposition—may increase, which may lead to a corresponding rebound in their abundance. In summary, bacterial communities appear to respond more actively to nitrogen addition and may be well-suited to environments with moderate to high nitrogen levels; fungi may be better adapted to high-carbon, low-nitrogen environments. The two groups may complement each other functionally and may jointly drive straw decomposition and nutrient cycling. This bacterial-versus-fungal duality echoes broader patterns reported in perennial berry systems: in blueberry, ericoid mycorrhizal fungi and oligotrophic taxa become increasingly dominant as plantation age advances and nitrogen availability declines, whereas copiotrophic bacteria respond rapidly to nutrient inputs [30]. The relative tolerance of fungal α-diversity to C/N manipulation observed here is therefore consistent with the conserved strategy of perennial berries, in which the fungal component mediates long-term organic-N acquisition rather than short-term fertilization responses.

Soil carbon and nitrogen nutrients, enzyme activity, and microbial communities interact and are mutually regulated, collectively sustaining biochemical cycles in the rhizosphere. Correlation analyses in this study indicate that bacterial diversity is positively correlated with soil nitrogen and active carbon fractions, while fungal diversity is negatively correlated with nitrogen fractions. It should be noted that these correlation results only represent associations rather than causal relationships and may reflect the intrinsic coupling patterns between microorganisms and soil environmental factors. Bacteria grow rapidly and depend on readily available nutrients; increased carbon and nitrogen levels may expand their ecological niche and enhance community diversity [31]. In contrast, fungi are predominantly oligotrophic, and high-nitrogen environments may inhibit their growth and reduce community diversity [32]. Microbial communities may indirectly influence nutrient transformation by regulating enzyme activity: bacteria and ascomycetes may enhance the activity of degradative enzymes and nitrogen-cycling enzymes, accelerating straw decomposition and nutrient release [33]. Basidiomycetes may enhance oxidase activity, promoting the transformation of recalcitrant carbon and humus formation [34]. Specifically, under the C/N = 20:1 treatment, the microbial community structure is well-balanced and enzyme systems function at a comparatively high level, which may form a positive feedback loop among substrates, the community, enzyme activity, and nutrients. This may improve the efficiency of straw nutrient transformation and contribute to the optimization of soil carbon and nitrogen pools as well as biological activity.

In summary, the application of straw in combination with nitrogen fertilizer to regulate the soil C/N ratio can effectively enhance nutrient levels, enzyme activity, and microbial diversity in the rhizosphere soil of *Lonicera caerulea*, with the best soil-improvement effect observed at a C/N = 20:1. From a global perspective, these results are timely: as haskap and other temperate perennial berries expand commercially beyond their traditional ranges, low-input, residue-based nutrient management offers a practical route to both soil carbon sequestration and reduced fertilizer dependence, a priority shared by perennial cropping systems worldwide [22]. These results can provide a theoretical basis for soil nutrient management in *Lonicera caerulea* cultivation. However, certain limitations remain: first, this study focused solely on the response of the rhizosphere soil environment and did not measure indicators such as plant growth, biomass, nutrient uptake, or fruit quality. Consequently, it is currently impossible to clearly determine the cascading effects of changes in the soil environment on plant phenotypes, and relevant agronomic recommendations still require support from experimental data at the plant level; second, this study was conducted as a controlled greenhouse pot experiment, which differs from actual field conditions. Factors such as field rainfall, soil heterogeneity, nutrient leaching, and the long-term decomposition dynamics of crop residue may all affect the field applicability of the results; further validation through long-term field experiments is required; and third, due to experimental constraints, the number of biological replicates was limited, and microbial analysis was conducted only at the phylum level; thus, there is room for improvement in the depth and statistical power of the study’s conclusions. Future research will focus on plant responses, field validation, and detailed microbial taxonomy to systematically refine the regulatory pathway linking “soil microenvironment—plant growth—fruit quality,” thereby providing a more comprehensive theoretical basis for the sustainable management of *Lonicera caerulea* forest soils.

## 4. Materials and Methods

### 4.1. Overview of Experimental Site

The experiment was conducted in plastic greenhouses at the teaching base of Jilin Agricultural University in Jilin Province. The experimental site has a temperate continental semi-humid monsoon climate, with an average annual temperature of 4.8 °C, a frost-free period of approximately 140–150 days, and an average annual precipitation of 522–615 mm, with rainfall concentrated primarily in the summer. The *Lonicera caerulea* variety tested was ‘E10’. The nitrogen fertilizer used in the experiment was urea (N, 45%).

### 4.2. Experimental Design

The experiment consisted of 7 treatments: control (CK), nitrogen fertilizer alone (N), corn stover alone (S), corn stover combined with nitrogen fertilizer (SN1, C/N = 10:1), (SN2, C/N = 15:1), (SN3, C/N = 20:1), (SN4, C/N = 25:1).

The experiment used pot-grown treatments. The pots had an upper diameter of 20.5 cm, a lower diameter of 14.5 cm, and a height of 18.5 cm. Each pot was filled with 2.5 kg of soil composed of field soil, peat moss, and perlite in a ratio of 2:1:1. The basic physicochemical properties of the mixed substrate were determined after thorough homogenization. The initial indicators were as follows: pH 6.34, soil organic matter 32.61 g·kg^−1^, total nitrogen 1.762 g·kg^−1^, available phosphorus 28.56 mg·kg^−1^, available potassium 29.3 mg·kg^−1^, and the initial C/N ratio was approximately 10.73.

This experiment adopted a fertilization principle based on a fixed carbon input, with 8 g of crushed corn stover added to each pot. The amount of nitrogen fertilizer (urea) added to each group was precisely calculated based on the predetermined C/N ratio to ensure the accuracy of each treatment ratio. The experiment used two-year-old *Lonicera caerulea* seedlings of uniform vigor and healthy growth for transplanting; one seedling was planted per pot, with three biological replicates for each treatment. The seedlings were transplanted in May and cultivated continuously for 5 months; samples were collected in October of the same year. During sampling, the topsoil was removed from each pot, and soil from the rhizosphere was collected using a soil auger. Approximately 10 g of rhizosphere soil was collected from each pot; plant root remnants, stones, and other impurities were removed, and the soil was thoroughly mixed to form an independent sample for subsequent analysis of soil microorganisms and soil environmental factors.

### 4.3. Soil Sample Analysis

Soil nutrients: Total nitrogen was determined using the Kjeldahl method, and alkali-hydrolyzable nitrogen was determined using the alkali-hydrolyzable nitrogen diffusion method [35]. Ammonium and nitrate nitrogen were determined using the KCl leaching method [36]. Dissolved organic carbon was determined using the potassium dichromate volumetric method [37]. Readily oxidizable carbon was determined using the 333 mmol·L^−1^ KMnO_4_ oxidation method. Dissolved organic carbon was determined using the deionized water leaching method. Olsen-P was determined using the 0.05 mol/L HCl–0.05 mol/L (1/2 H_2_SO_4_) method. Available potassium is determined using the NH_4_OAc extraction–flame photometry method.

Soil Enzyme Activity [38]: Urease was determined using the sodium phenolate–sodium hypochlorite colorimetric method; acid phosphatase was determined using the disodium phenylphosphate colorimetric method; catalase was determined using the potassium permanganate titration method; sucrase was determined using the 3,5-dinitrosalicylic acid colorimetric method, and β-glucosidase was determined using the p-nitrophenol colorimetric method.

Soil microbial community diversity: Total microbial DNA was extracted from the soil, and target fragments of bacterial 16S rRNA and fungal ITS were amplified. After library construction, the samples were sent to Shanghai Meiji Biotechnology for high-throughput sequencing on the Illumina MiSeq platform.

### 4.4. Data Analysis and Processing

Experimental data are presented as the mean ± standard error of three biological replicates. Data were statistically analyzed and organized using Microsoft Excel 2019. SPSS Statistics 19 (IBM Corp., Armonk, NY, USA) was used to perform statistical analysis of the measured data. First, the Shapiro–Wilk test and Levene’s test were used to verify the normality of the data and the homogeneity of variances, respectively. After confirming that the assumptions for parametric tests were met, a one-way analysis of variance (ANOVA) was conducted, followed by Duncan’s multiple range test (*p* < 0.05). GraphPad Prism 10.1.2 was used for data visualization. Quality control of soil rhizosphere microorganisms, OTU clustering, species annotation, alpha/beta diversity analysis, and differential species analysis were performed on the Majorbio Cloud Platform (https://www.majorbio.com, Shanghai, China). Association analysis between microorganisms and soil environmental factors was conducted using the R software version 4.4.1 (R Core Team, Vienna, Austria; https://www.r-project.org/), and community analysis charts were generated using both platform tools and R.

## 5. Conclusions

Currently, there remains a relative lack of research, both domestically and internationally, on the application of organic fertilizers in *Lonicera caerulea* cultivation systems. In this study, by fixing the amount of straw carbon input and gradually adjusting the nitrogen fertilizer application rate to establish different C/N ratio treatments, we conducted a systematic comparative analysis of nutrient composition, enzyme activity, and microbial community characteristics in the rhizosphere soil of *Lonicera caerulea* plants. The study confirmed that a C/N ratio of 20:1 (SN3) is the ideal stoichiometric point for maximizing the synergistic effects of soil carbon and nitrogen accumulation and microbial community diversity. This ratio enables efficient coupling of straw decomposition and nutrient transformation, effectively reduces the risk of nutrient loss, and fully leverages the synergistic soil-improving effects of co-application of straw and nitrogen fertilizer. Based on the results of the pot experiments, a fertilization regimen with a C/N ratio of 20:1 is recommended for cultivating *Lonicera caerulea* seedlings to maximize the soil-improving effects of combined carbon and nitrogen application on the rhizosphere.

The findings of this study identify the optimal C/N regulation threshold for the application of nitrogen fertilizer in conjunction with straw return during the seedling stage of *Lonicera caerulea* cultivation, providing a quantitative basis for soil improvement and nutrient management during the *Lonicera caerulea* seedling stage. It holds significant practical guidance for promoting the resource utilization of straw in orchards, enhancing nitrogen fertilizer use efficiency, reducing nitrogen loss to the environment, and achieving sustainable agricultural management of forest land soils.

This study verified the ameliorative effects of co-application of crop straw and nitrogen fertilizer only under potted conditions; while the optimal C/N ratio identified here was applied to *Lonicera caerulea* cultivation in open fields, actual results may vary due to the interaction between field soil conditions and seasonal climate. Follow-up studies will include multi-year field plot trials and dynamic monitoring during the growing season to establish the optimal straw-nitrogen fertilizer application scheme tailored to the on-site conditions of orchards, thereby providing a technical basis for standardized *Lonicera caerulea* seedling propagation and sustainable soil fertility management in forested areas.

## Figures and Tables

**Table 1:** Effects of different C/N ratios on soil nitrogen and carbon fractions.

Treatment	SOM (g·kg^−1^)	TN (g·kg^−1^)	AN (mg·kg^−1^)	ROC (g·kg^−1^)	DOC (mg·L^−1^)	NH_4_^+^-N (mg·kg^−1^)	NO_3_^−^-N (mg·kg^−1^)
CK	54.91 ± 2.44 b	0.33 ± 0.01 d	125.40 ± 1.74 d	1.40 ± 0.25 c	200.45 ± 19.78 d	63.13 ± 3.72 d	77.70 ± 1.85 e
S	55.55 ± 6.33 b	0.43 ± 0.03 cd	136.53 ± 0.06 c	1.43 ± 0.26 c	296.12 ± 30.23 bc	72.42 ± 1.05 cd	90.31 ± 5.58 d
N	63.98 ± 3.98 a	0.44 ± 0.12 bcd	142.57 ± 2.38 bc	1.44 ± 0.14 c	258.14 ± 20.67 c	74.06 ± 5.50 c	92.92 ± 5.55 d
SN1	54.27 ± 2.98 b	0.50 ± 0.08 bc	145.08 ± 3.69 ab	1.79 ± 0.21 bc	297.72 ± 28.30 bc	82.28 ± 4.72 bc	128.98 ± 0.83 c
SN2	54.66 ± 7.08 b	0.59 ± 0.12 ab	145.67 ± 2.75 ab	1.54 ± 0.47 c	343.07 ± 41.14 ab	90.72 ± 7.99 b	157.26 ± 1.92 b
SN3	66.53 ± 4.20 a	0.70 ± 0.07 a	150.68 ± 7.48 a	2.39 ± 0.07 a	379.06 ± 20.74 a	111.64 ± 7.19 a	237.04 ± 10.97 a
SN4	60.66 ± 2.24 ab	0.54 ± 0.05 bc	145.20 ± 2.36 ab	2.12 ± 0.12 ab	363.33 ± 35.88 a	86.21 ± 4.47 b	133.86 ± 3.97 c

Note: Different lowercase letters within the same column indicate significant differences among treatments (*p* < 0.05).

**Table 2:** Analysis of enzyme activities in the rhizosphere soil of *Lonicera caerulea*.

Treatment	URE Activity (mg·g^−1^·24 h^−1^)	SUC Activity (mg·g^−1^·d^−1^)	CAT Activity (mL·g^−1^·h^−1^)	ACP Activity (mg·g^−1^·24 h^−1^)	β-GC Activity (nmol·g^−1^·h^−1^)
CK	0.66 ± 0.07 c	2.69 ± 0.59 c	62.29 ± 0.29 c	0.74 ± 0.11 c	84.41 ± 3.05 e
S	0.79 ± 0.08 ab	2.95 ± 0.89 bc	63.11 ± 0.85 bc	0.86 ± 0.09 bc	94.69 ± 2.60 d
N	0.69 ± 0.03 bc	2.87 ± 0.42 bc	63.10 ± 0.12 bc	0.74 ± 0.10 c	93.96 ± 4.26 d
SN1	0.80 ± 0.07 ab	3.86 ± 0.30 ab	63.60 ± 0.10 b	0.88 ± 0.09 bc	107.65 ± 3.02 c
SN2	0.81 ± 0.01 ab	4.04 ± 0.74 a	63.75 ± 0.71 ab	0.90 ± 0.19 bc	133.63 ± 3.84 b
SN3	0.90 ± 0.07 a	4.64 ± 0.34 a	64.50 ± 0.07 a	1.26 ± 0.15 a	143.69 ± 2.51 a
SN4	0.80 ± 0.07 a	4.35 ± 0.59 a	63.94 ± 0.22 ab	0.99 ± 0.10 b	135.08 ± 1.09 b

Note: Different lowercase letters within the same column indicate significant differences among treatments (*p* < 0.05).

## Data Availability

The original contributions presented in this study are included in the article. Further inquiries can be directed to the corresponding author.
